# *In vitro* secretion of zymogens by bovine pancreatic acini and ultra-structural analysis of exocytosis

**DOI:** 10.1016/j.bbrep.2015.12.009

**Published:** 2015-12-23

**Authors:** Sivalingam Jayaveni, Kamaraj Nithyanandham, Chellan Rose

**Affiliations:** Department of Biotechnology, CSIR – Central Leather Research Institute, Chennai 600020, Tamil Nadu, India

**Keywords:** Bovine acinar, SEM, TEM, Pancreatic enzymes, Exocytosis

## Abstract

The aim of this study is to establish a bovine pancreatic acinar cell culture model with longer viability and functionality. The cells could be maintained in a functional state for upto 20 days with normal morphology. Cells were positive for amylase as observed by immunofluorescence staining. Acinar cells are spherical and range about 2–3 µm in diameter. The porosome formed by exocytosis and heterogenous enzyme granules of size ranging 100–300 nm were seen on the surface of cells by electron microscopy. The activity of the enzymes was high on day 15 and the activity profile of the enzymes is in the order: protease>lipase>amylase and the enzymes were identified by SDS-PAGE. Long-term culture of bovine pancreatic acini could be useful in studying the pathogenesis of pancreatitis. Since the bovine genome shares about 80% identity with the human genome, the cells derived from bovine pancreas can be engineered and used as a potential xenotransplant to treat conditions like pancreatitis as the tissue source is abundantly available.

## Highlights

• Acinar cells are functional unit of pancreas.
• *In vitro* release of enzyme granules by exocytosis are well demonstrated.
• Amylase shares 86% protein identity with that of human.
• Acinar culture showed longer viability upto 20 days with normal cellular function.
• Useful culture model in understanding the acinar cell biology and physiology.

## 1 Introduction

The pancreatic acinar cells are highly specialized cells which have the greatest rate of protein synthesis among all cells in the body [1] and constitute about 80% of pancreas. The major function of the acinar cell is the synthesis, storage and secretion of digestive enzymes *viz.*, proteases, lipase, amylase, ribonucleases and elastase so as to catalyze the hydrolysis of food constituents into absorbable forms. These enzymes are secreted as proenzymes and are packed in secretory granules [SV] as inactive precursors and released by exocytosis, a process which involves the movement of the secretory granule to the apical surface and recognition of a site on the plasma membrane for fusion and release. The proenzymes are activated when they enter the duodenum by enterokinase. The enterokinase, first activates trypsinogen by removing an N-terminal hexapeptide fragment of the molecules (Val–Asp–Asp–Asp–Lys] by hydrolysis [2], [3], [4]. Thus activated trypsin sequentially activates other proenzymes. The premature activation of these enzymes inside the pancreas causes a pathological condition called pancreatitis, the autodigestion of pancreas.

The mechanisms involved in the development and course of this disease remain obscure. To study the molecular mechanisms involved in such pathological conditions, a number of animal models like rat [5], [6], [7], [8], canine [9], hamster [10], porcine [11] and human [12] have been developed. Over the last few years, several methods have been described for culture of pancreatic acinar cells which produced cultured cells having vastly different characteristics [13], [14], [15].

Despite variation in the dissociation protocols, culture media and nutrient supplements, it has not been possible to maintain primary cultures of pancreatic acinar cells for more than 4 days [5]. The reason may be that the zymogens are activated within the pancreatic acinar cells [16], [17] and the systemic effects of these active pancreatic enzymes result in widespread tissue damage. The cellular signalling cascade mechanisms of pancreatic acinar cells have been reported [18]. Agents that efficiently inhibit intracellular zymogen activation and block the activity of released enzymes may prevent acinar cell damage. It has been reported earlier that high or low pH, makes the zymogen granules fragile resulting in lysis of the cells [19]. The release and activation of protease, which occurs during collagenase digestion of rat, porcine, canine, and human pancreas, can be dramatically reduced if the digestion is carried out in a solution containing 10% BSA [20]. Moreover, albumin possesses the capacity to act as a scavenger of oxygen free radicals [21]. Since the acinar cells are loaded with proteolytic enzymes, they are prone to lysis and cell death. As a consequence, this culture is short lived and usually does not last over 20 h [22]. Cell culture model with longer viability could be useful in studying the normal physiology and pathophysiology like pancreatitis, cancer *etc.*, Therefore, with this background and warranted requirements, we made an attempt to isolate and characterize the bovine pancreatic acinar cells for their identity and long term culture using supplements without allowing any change in their normal cellular physiology.

## 2 Materials and methods

### 2.1 Primary culture of bovine pancreatic acinar cells

The pancreas was collected afresh on ice from local abattoir and processed within 2 h. The cells were isolated by the method of Bruzzone et al. [5]. Briefly, the procedure involved chopping of fat-trimmed bovine pancreatic tissue followed by digesting it in a dissociation medium containing collagenase III (1 mg/ml) in Krebs-Ringer buffer with 5% BSA and incubated for 15 min with constant shaking until homogenous solution was obtained. To this, 5 ml of fresh RPMI 1640–Hepes–Fetal bovine serum was added and centrifuged at 500×*g* for 30 s. The cell pellet obtained was washed twice followed by centrifugation. Cells were resuspended in fresh medium with supplements (1 nM Epidermal growth factor, 5% BSA), 10% fetal bovine serum and antibiotics (100 U/ml Penicillin and 100 µg/ml Streptomycin) and incubated at 37 °C with 5% CO_2_ and the cells were maintained as suspension culture in a same medium throughout the culture period.

### 2.2 Culture maintenance

Cells were seeded in a 24-well culture plate at a density of 1×10^5^ cells/ml and incubated at 37 °C with 5% CO_2_. The viability of acinar cells was monitored at specific time intervals using trypan blue dye exclusion test and also by fluorescence staining using Acridine orange/Ethidium bromide. The morphology of the acinar cells was examined periodically under inverted phase contrast microscope (Leica DMI-IL LED, Germany). The cells were harvested on day 5 for characterization studies.

### 2.3 Immunofluorescence staining

Acinar cells were fixed with 1% formalin for 10 min after a brief wash with PBS and then washed again with PBS thrice. After fixation, cells were incubated with amylase antibody raised against human (H-281, Santa Cruz, USA) at a concentration of 5.0 μg/ml in PBS with 1.5% blocking serum for 60 min and washed thrice with PBS at 5 min interval to remove unbound antibodies. Then the cells were incubated in dark for 45 min with Goat anti-rabbit IgG–R conjugated secondary antibody (5 μg/ml) in PBS with 1.5–3% blocking serum. The cells were treated with serum in place of primary antibody for negative control. After incubation, the coverslip was mounted with aqueous mounting medium and examined under trinocular fluorescence microscope (Leica DMI-IL LED, Germany).

### 2.4 Scanning electron microscopic (SEM) analysis

Surface morphology of the acinar cells was visualized by using High Resolution Scanning Electron Microscope (FEI Quanta FEG 200, USA) to obtain the high resolution images. Cultured acinar cells were harvested on day 5 separated after centrifugation at 500×*g* for 5 min were re-suspended in phosphate buffer (pH 7.4). The cells were then fixed with buffered formalin and post fixed with 1% Osmium tetroxide in phosphate buffer (pH 7.4). After dehydration in graded series of ethanol from 10% to 99.9% the cells were coated with gold and analyzed under High Resolution Scanning Electron Microscope by operating it at an accelerating voltage of 10–20 kV at different magnification levels.

### 2.5 Transmission electron microscopic (TEM) analysis

Cells were harvested on day 5 and fixed with 2.5% glutaraldehyde buffered with 0.1 M sodium cacodylate buffer (pH 7.4) for 2 h and centrifuged at 500×*g* for 5 min. Then the cells were post fixed with 1% osmium tetroxide in the same buffer for about 1 h. After dehydration in a graded series of ethanol for 15 min each, the cell pellet was embedded in Epon 812 resin and ultra-thin sections were obtained using ultra-thin microtome and mounted on copper grids. The sections were stained with 3% aqueous uranyl acetate and Reynolds lead citrate for 5 min and 3 min respectively. The ultrastructure of the acinar cells was observed under transmission electron microscope (JEOL-1010, USA).

### 2.6 Biochemical characterization of acinar cells

The culture medium was collected at different intervals of culture period *i.e.*, on day 5, 10, 15 and 20, centrifuged at 500×*g* for 10 min and the supernatant was collected and used for assaying the activities of enzymes. The activity of the enzymes was determined using standard protocols to study the functional characteristics of the cells as the enzyme secretion is the main function of the acinar cells.

(1) *Amylase assay –* Amylase activity of the culture supernatant was measured according to the method of Miller [23] with slight modification using dinitro salicylic acid (DNS) reagent. A 0.2 ml of enzyme sample was made up to 0.5 ml with water and incubated with 0.5 ml of 1% (w/v) starch solution for 15 min followed by addition of 1% (w/v) DNS solution and boiled for 10 min to terminate the reaction. Amylase hydrolyzes 1, 4-glycoside linkages at every other junction between carbon 1 and oxygen of the starch molecule and release maltose. The increase in concentration of reducing sugar maltose was measured at 540 nm. Maltose is used as a standard.
(2) *Lipase assay –* Lipase activity was measured spectrophotometrically as described by Lehner and Verger [24]. About 50 μl of the enzyme sample was incubated with 950 μl of p-nitrophenyl laurate for 15 min. The reaction was arrested by the addition of 2 ml of acetone and the absorbance of released p-nitrophenol was read at 410 nm. p-Nitrophenol is used as a standard.
(3) *Protease assay –* The proteolytic activity was determined by the method of Kunitz [25] using casein as substrate. Prior to the assay, the enzyme was activated by incubating the enzyme solution with calcium chloride to a final concentration of 0.1 M [17]. 0.2 ml of enzyme was made up to 1 ml using 0.1 M phosphate buffer pH 7.4 and incubated for 20 min with 1 ml of 1% casein in the same buffer and the reaction was terminated by the addition of 3 ml of 10% trichloroacetic acid (TCA). A control experiment was conducted in the same manner but the TCA was added prior to the substrate. The absorbance of the TCA soluble fraction was read at 280 nm. Tyrosine is used as standard to calculate the activity of protease.

### 2.7 Electrophoresis and zymography

SDS-PAGE of the enzymes was performed according to the method of Laemmli [26] using 10% gel. The molecular weight of the enzyme(s) was determined by using a medium range protein molecular weight marker (97.4–14.3 kDa, GeNei, Bangalore). A zymogram of CaCl_2_ treated crude enzyme was accomplished on separating gel, containing 0.1% gelatin as the substrate. Following electrophoresis, the gel was washed successively 2.5% (v/v) triton X-100 twice to remove SDS. The gel was then incubated with developing solution (Tris buffer, pH 7.4) at 37 °C for 18 h. Later, the gel was stained with coomassie brilliant blue R250 for 30 min and destained overnight to reveal zones of substrate lysis. The intensity of the zones was measured using BIO RAD Gel Doc EZ Imager.

## 3 Results

### 3.1 Cell culture and proliferation

**Fig. 1:**
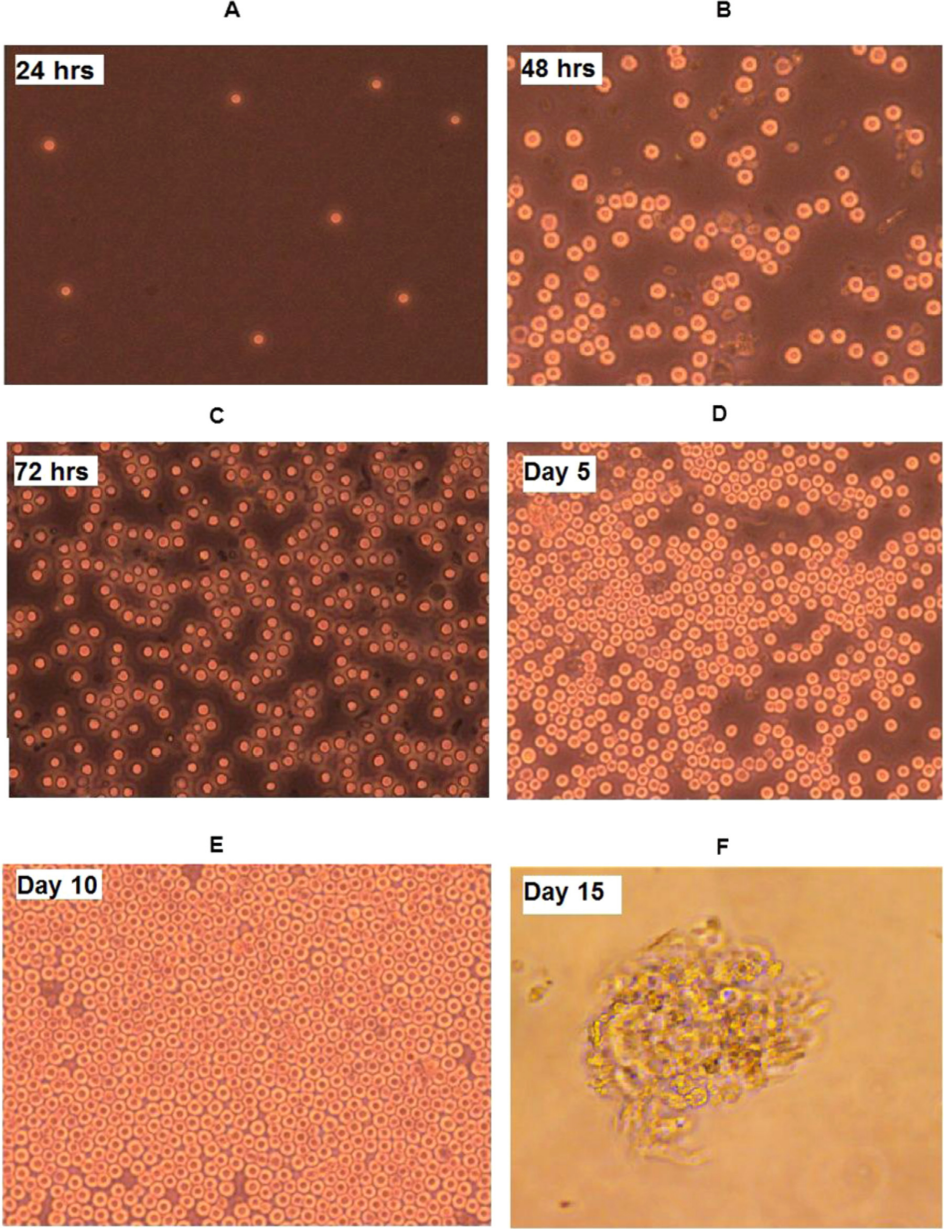
Phase contrast images of isolated pancreatic acinar cells at different culture periods (A) Freshly isolated acinar cells; (B, C, D) Proliferating acinar cells; (E) Monolayer formation; (F) Acini cluster, Mag. 40×.

Acinar cells isolated from bovine pancreas by collagenase digestion appeared spherical in shape (Fig. 1A) when visualized under phase contrast microscope. Throughout the culture period the cells were healthy with intact cell membrane showing no apoptotic/necrotic blebs. The cells were proliferative (Fig. 1B and C) from day 2 onwards, attracted towards each other, then started to aggregate (Fig. 1D) and formed a monolayer (Fig. 1E). At this stage, nuclei were seen clearly due to the pressure created on the cell membrane by crowding of cells. After a passage, the cells formed clusters (Fig. 1F) of different sizes and shapes. The size of the cluster increased with increase in culture period and retained structural organization as found in the tissue (data provided in Supplementary material).

### 3.2 Cell viability

**Fig. 2:**
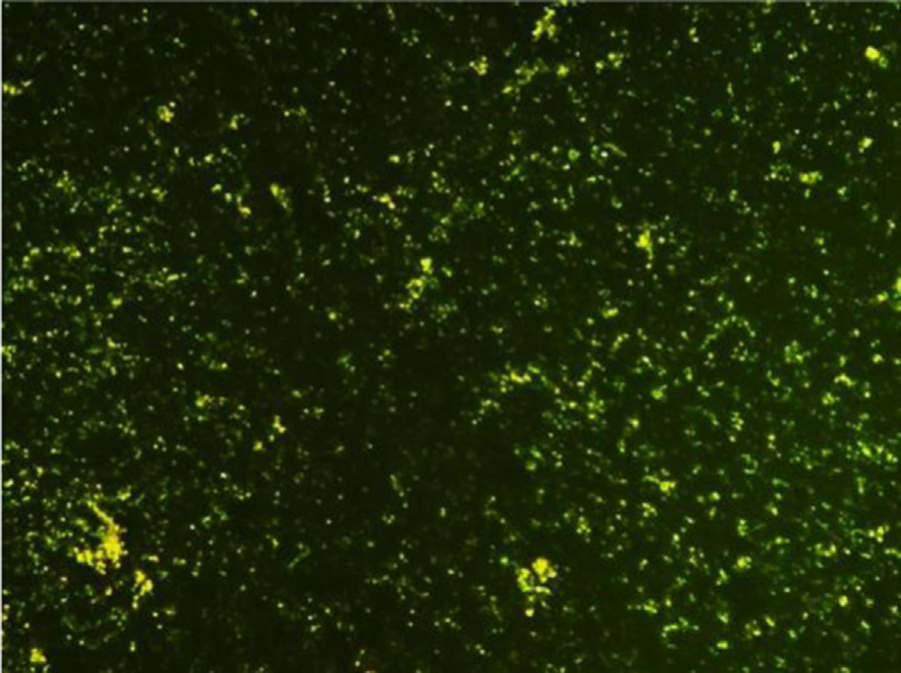
Viability of acinar cells: fluorescent labelled acinar cells; green fluorescence – acridine orange, Mag: 4×.

The cells were viable and proliferative throughout the culture period in an unchanged medium as observed from trypan blue exclusion test. The rate of proliferation increased with increase in incubation time (Table 1). The viability of acinar cells was also observed by staining with acridine orange and ethidium bromide, where the former fluorescent dye penetrates into intact cells and stains DNA of live cells to emit green fluorescence while the latter does not penetrate intact cell membranes; thus staining dead cells to give red fluorescence. When observed under a fluorescent microscope, all the confluent cells emitted green fluorescence (Fig. 2) indicating that the cells were viable.

**Table 1:** Total cell count of bovine pancreatic acinar cells at specific time intervals.

**Incubation time (in days)**	**Cell count (cells /ml)**
**Day 5**	1.12×10^6^
**Day 10**	1.89×10^6^
**Day 15**	2.62×10^6^
**Day 20**	2.83×10^6^

### 3.3 Immunofluorescence staining

**Fig. 3:**
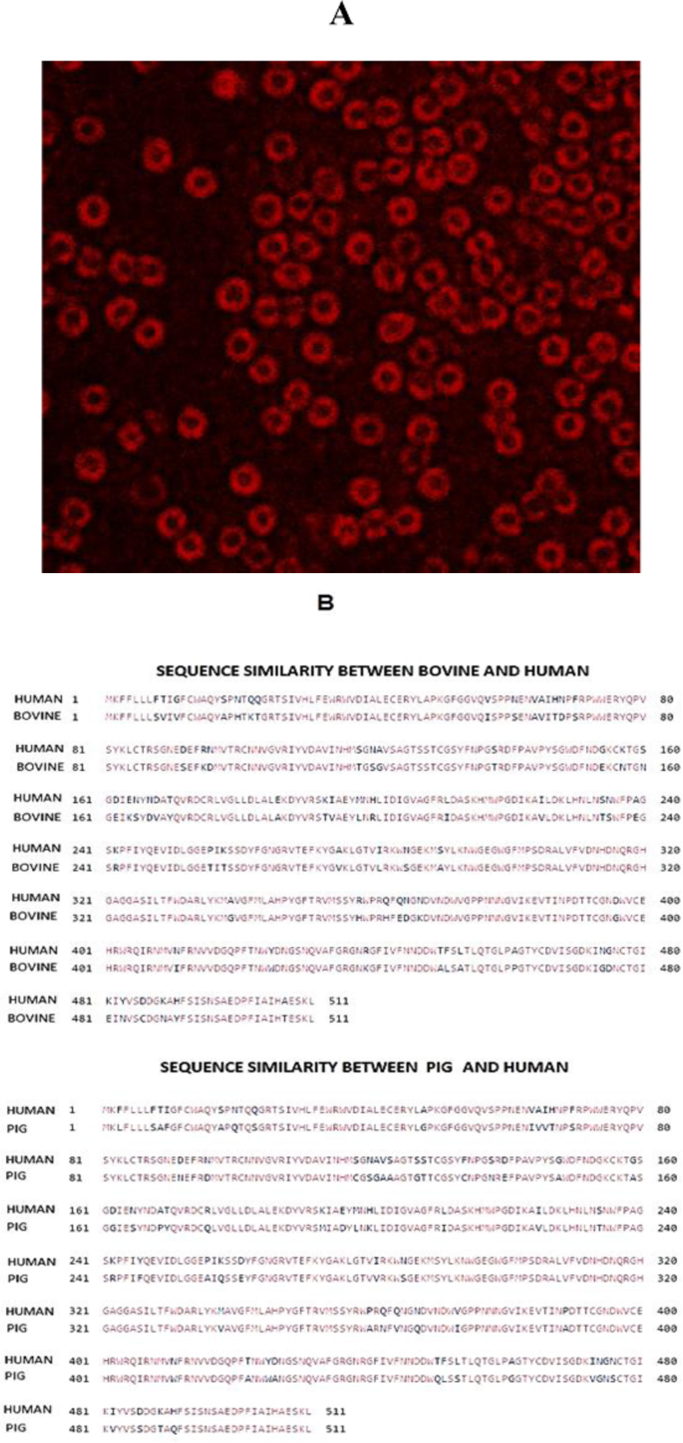
Immunofluorescence and BLAST (A) Immunofluorescence staining – Cytoplasmic localization of amylase; Mag: 40×; (B) Sequence similarity search results of amylase 2B proteins by BLASTP (i) Bovine and Human amylase 2B (ii) pig and human amylase 2B.

Cellular localization of amylase in acinar cells was done by immunofluorescence staining using antibody against amylase. The image is presented in Fig. 3A. All the cultured bovine acinar cells emitted red fluorescence after binding to the human amylase antibody. This observation confirmed the purity of acinar culture. Protein sequence similarity search was done using protein-protein Basic Local Alignment Search Tool (blastp)” for amylase 2B protein of bovine (Uniprot # F1MJQ3) and human (Uniprot # P19961). The percentage of identity between those two proteins was found to be 86% (Fig. 3B). Since porcine was so far thought to be closer to human and tissues from pigs were used as transplants. Therefore we have also checked the sequence similarity between human and pig amylase 2B (Uniprot #I3LAV8) proteins using the same tool (Fig. 3B). Surprisingly, the pigs also share 86% identity with human amylase 2B.

### 3.4 Scanning Electron Microscopy (SEM)

**Fig. 4:**
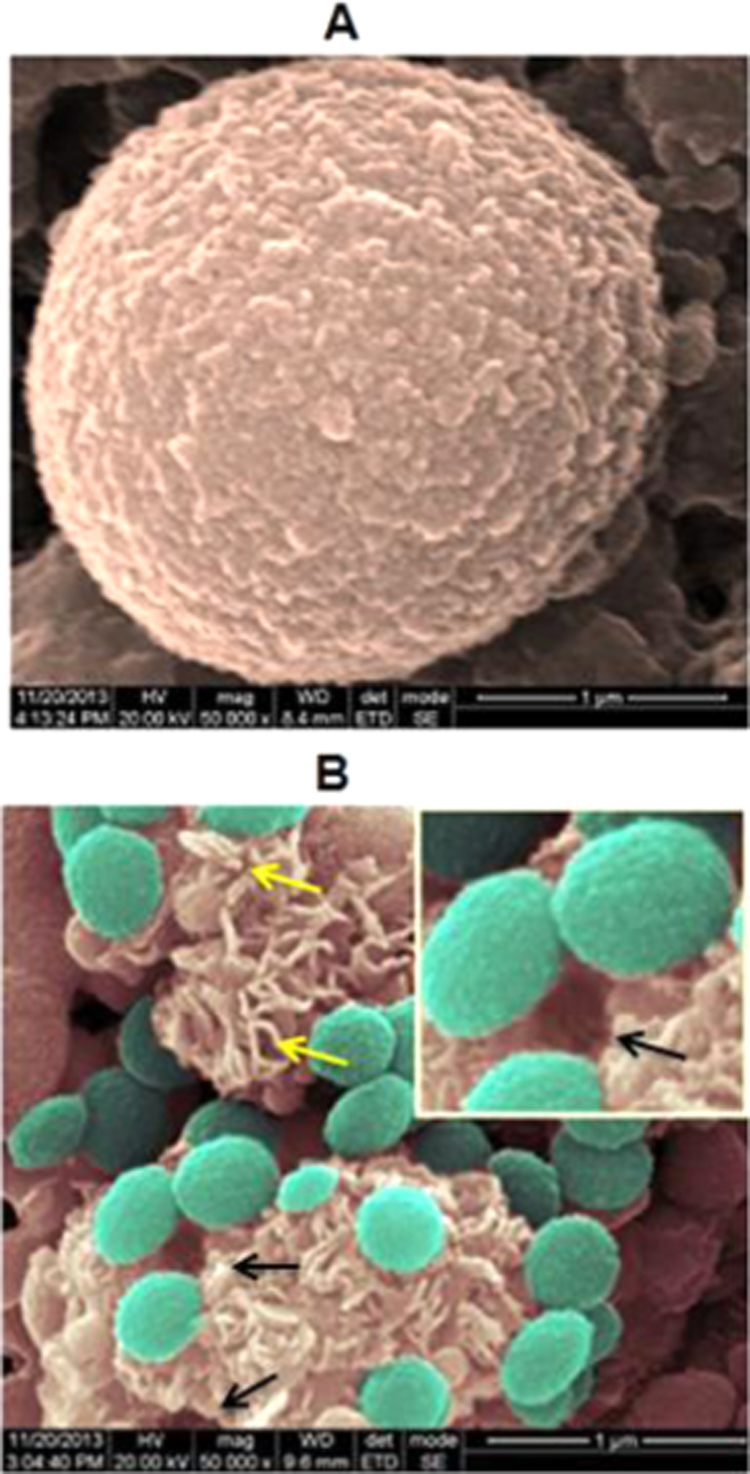
False coloured SEM image of acinar cells. (A) Acinar cell in a resting state, mag: 50,000× (B) exocytosis of zymogens (cyan). Black arrow indicates the fusion pore; and a magnified image of the same is given in the right hand top corner of the image, mag: 50,000×; yellow arrow indicates microvilli.

To observe the detailed surface morphology, the bovine pancreatic acinar cells were visualized under High Resolution – Scanning Electron Microscope. To present the morphological features more clearly, the SEM images are false-coloured. As observed from Fig. 4A, the resting acinar cell was spherical in shape with smooth surface similar to the phase contrast microscopic visualization (Fig. 1A). The size of the acinar cell ranges from 2 to 3 µm. Few cells exhibited exocytosis, a rare event to be captured on a scanning electron microscope. Providentially, this may be due to fixation of the cells during the process of exocytosis while preparing the samples for analysis. Changes were observed on the surface of the cells during exocytosis, as some microvilli projections were seen on the surface (Fig. 4B). Fusion pore or porosome, a small pit like structure formed as a result of exocytosis/enzyme secretion was seen at the site of release and indicated by arrow in Fig. 4B. The released granules are non-homogenous in nature with different sizes and shapes.

### 3.5 Transmission Electron Microscopy

**Fig. 5:**
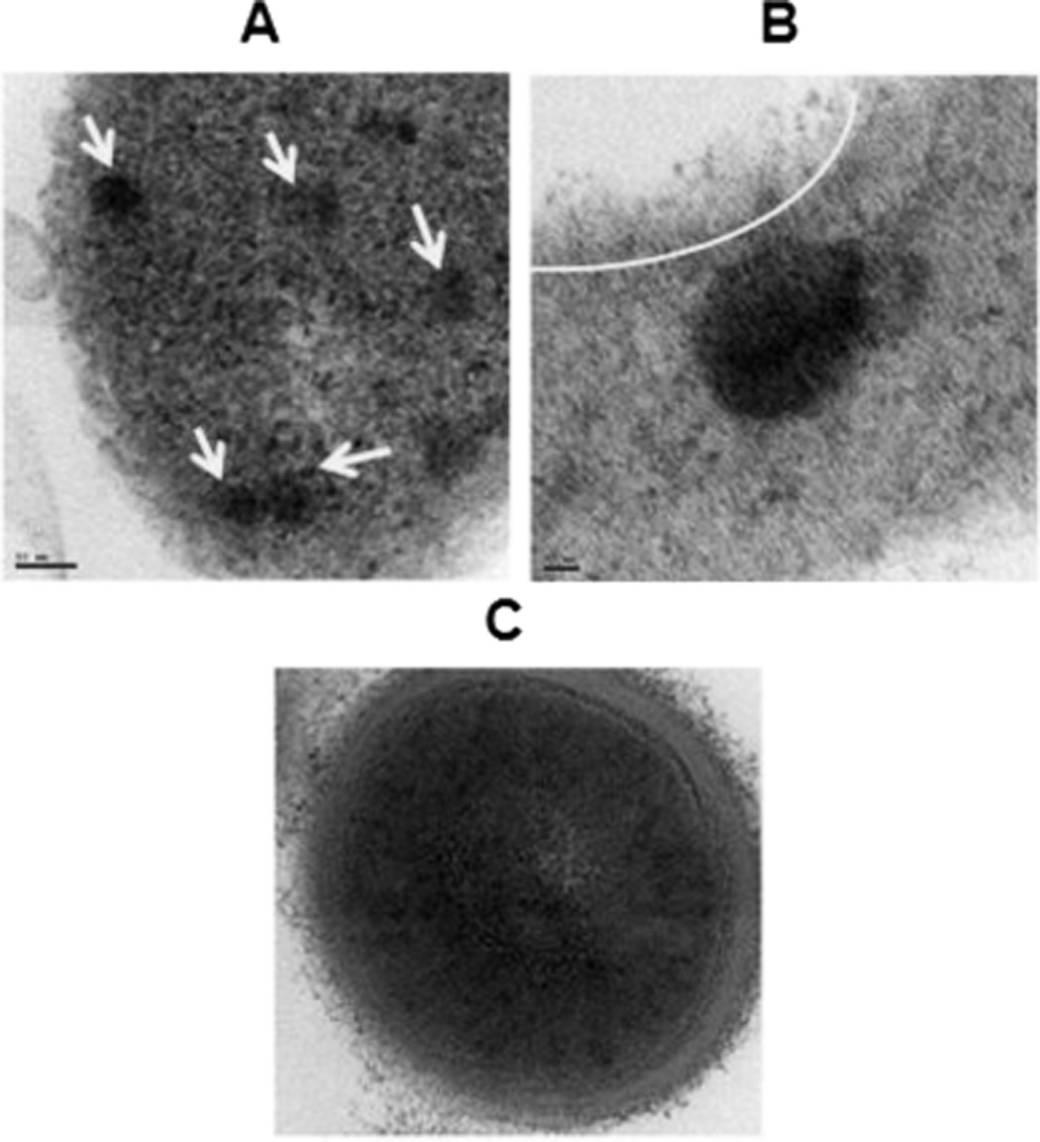
TEM image of pancreatic acinar cell. (A) Secretory granules ready for release and arrows indicates the docking vesicles, scale bar: 50 nm; arrows indicates the secretory granules. (B) Fused secretory granule with cell membrane. The arc indicates the fusion pore, scale bar: 20 nm. (C) Magnified image of Secretory granule showing the lipid bilayer;

The ultra-structure of the acinar cells were observed under a transmission electron microscope and photographed. A few steps involved in the exocytosis process were captured in TEM and presented as Fig. 5 A–C. Fig. 5A shows the presence of active secretory granules dispersed in cytoplasm of the acinar cell and these are indicated by arrows. The secretory granules, which appear as dark patches, were observed to be approaching the plasma membrane of the cell for docking, the first stage of exocytosis. In Fig. 5B the arc indicates the formation of fusion pore/porosome in the plasma membrane, due to priming of the secretory granule to the membrane, which is the second stage of exocytosis. Microvilli projections seen in the plasma membrane of TEM image authenticate the data obtained in the SEM. The closer view of the independent secretory granule is presented in Fig. 5C. As observed from the image, the secretory granule is spherical with double layered membrane and filled with granules.

### 3.6 Biochemical characterization

**Fig. 6:**
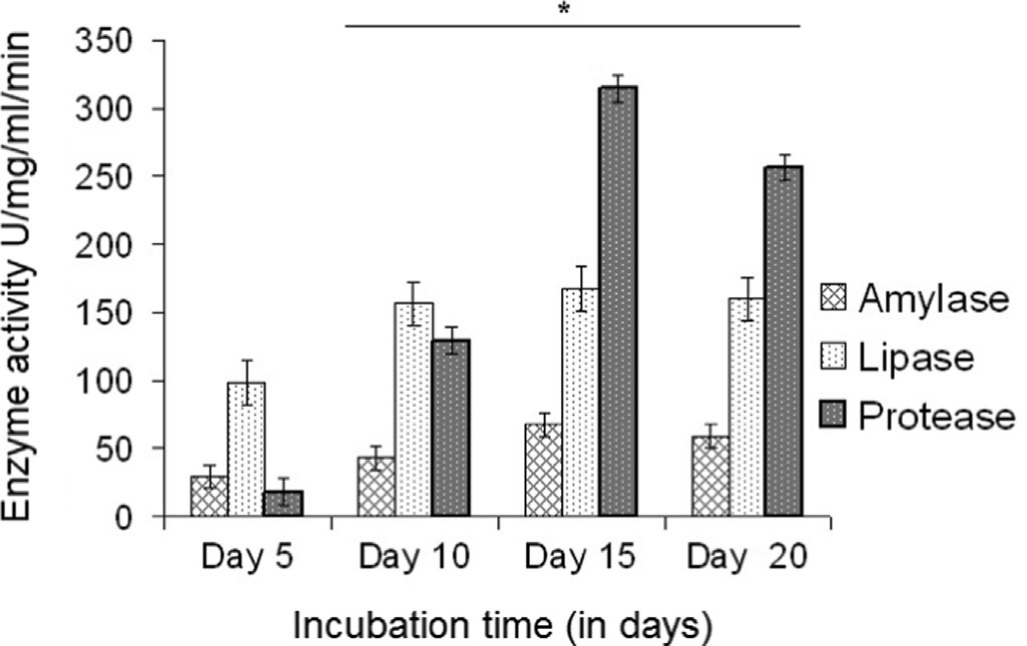
Graph depicting the activities of enzymes secreted by acinar cells on day 5, 10, 15 and 20. Results are mean±SD from ≥3 determinations for each time point; *P<0.05 compared with cells at day 5.

Amylase, lipase and protease activities determined at 5 days interval for upto 20 days are presented in Fig. 6A. The activity of all the three enzymes gradually increased till 15 days of incubation. Later, on day 20, only protease showed a 15% decrease while the activity of lipase and amylase remained almost unchanged. This may be due to depletion of nutrients in the culture medium. On day 5, the lipase activity was found to be higher than that of the other two enzymes. Activities of lipase and amylase gradually increase with incubation time. The activity of protease was comparatively low during the 5th day and a sudden increase in its activity was observed on day 15. This is the first report on protease activity of pancreatic acinar cells. The results obtained from enzyme assays indicate that the pancreatic acinar cells are viable and functionally active as well.

### 3.7 Electrophoresis and zymography

**Fig. 7:**
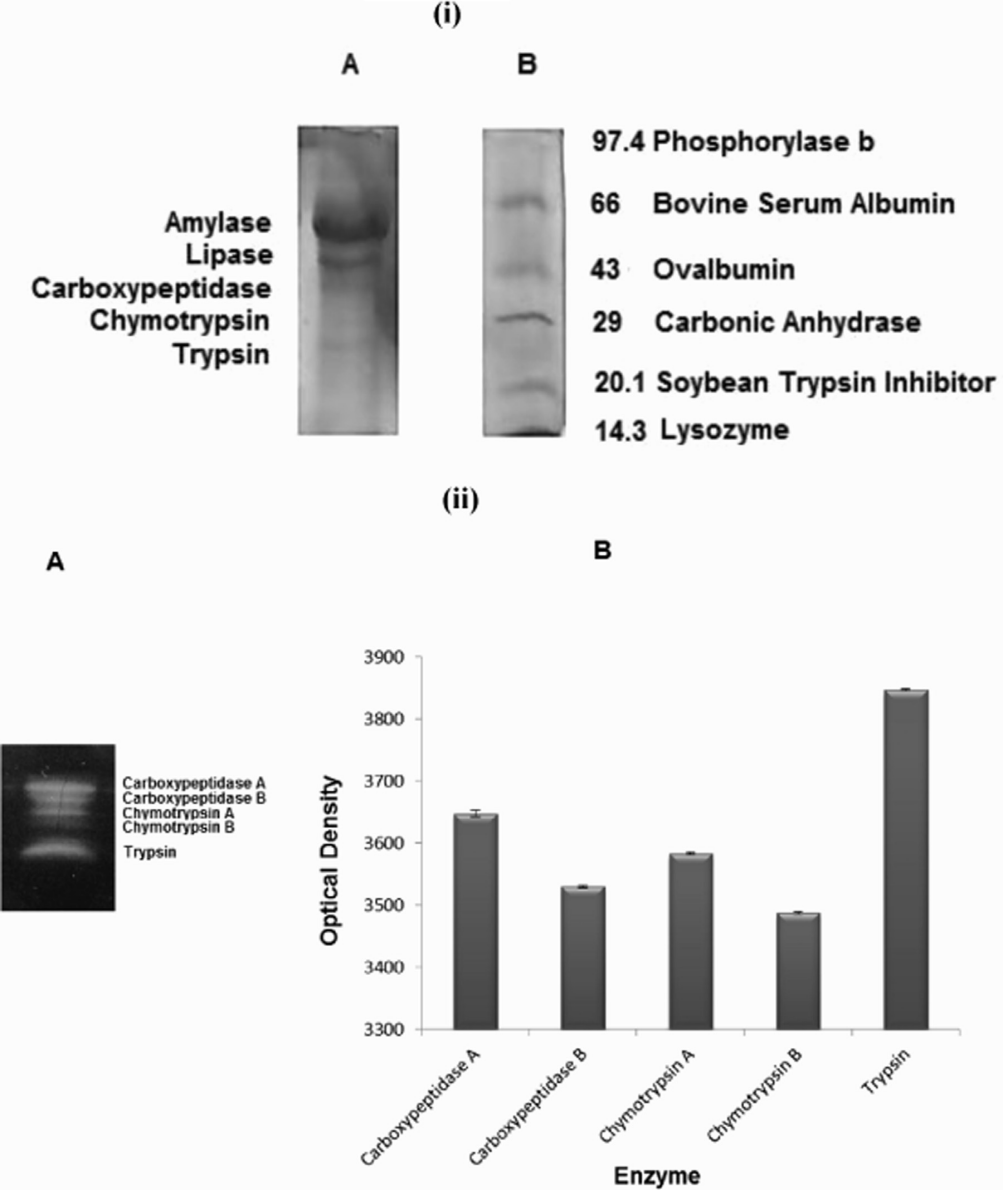
(i) Electrophoretic pattern of the pancreatic enzyme and molecular weight marker (A) Lane 1: enzyme sample, the bands are identified as (a) Amylase (b) Lipase (c) Carboxypeptidase (d) Chymotrypsin (e) Trypsin (B) Molecular weight marker (range 14.3 kDa–97.4 kDa); Lane: 2 Molecular weight marker. (ii) Zymogram of the pancreatic enzymes (A) Five clear bands were observed. Two of the proteases appeared in a dimeric pattern showing the isoforms. Based on the molecular weight the enzymes were found to be (a) Carboxypeptidase A (b) Carboxypeptidase B (c) Chymotrypsin A (B) Intensity of individual enzymes; Mean±SD *n*=5.

The electrophoretic patterns of secretory proteins/enzymes of acinar cells are given in Fig. 7(i). The molecular weight of the proteins was determined by comparing their mobility with that of the standard molecular weight markers. The three major enzymes of bovine pancreas thus identified are marked in the figure. The secretion of proteolytic enzyme in the culture medium was confirmed further by the protein cleavage zones in the gelatin incorporated gel by proteases secreted by acinar cells. This substantiated the secretion of enzymes and their digestive nature. The molecular weight of these proteolytic enzymes thus secreted ranged from 24 kDa to 35 kDa. A zymographic study revealed the presence of isoforms of carboxypeptidase and chymotrypsin which is confirmed by the occurrence of dimeric structures (Fig. 7(ii) A). The band intensity of these enzymes were determined and illustrated in Fig. 7(ii) B. The intensity of trypsin was found to be the highest followed by carboxypeptidase A, chymotrypsin A, carboxypeptidase B and then chymotrypsin B, thus re-establishing the fact that trypsin contributes more for the proteolytic activity.

## 4 Discussion

Exocrine pancreatic dysfunction includes acute pancreatitis, chronic pancreatitis, autoimmune pancreatitis, pancreatic insufficiency and pancreatic neoplasia. To study such pathogenesis, a suitable *in vitro* model is necessary. Hence this study was aimed at having a simplified procedure for culturing bovine pancreatic acinar cells for longer duration to study the functional characteristics of cells *in vitro.*

A lot of research has been carried out on pancreatic acinar cell culture on porcine/rodent models which involved very tedious processes [27], [13], [28]. Therefore, we in this study isolated and cultured pancreatic acinar cells from bovine pancreas by a simple procedure described by Bruzzone et al. [5] with slight modifications. Freshly isolated cells formed a monolayer after a few days of isolation; and the cells started to aggregate and formed clusters after few passages as the term ‘acinus', meaning “cluster of cells” suggests. The bovine pancreatic acinar cells showed similarity in morphology with rodents, murine, porcine and human [27], [11], [12], [29].

The literature on maintenance of rat and murine pancreatic acinar cells *in vitro* indicates that the cells remain viable and functional for only 4–12 days [14], [18]. In this investigation, we were able to keep the cells viable and functional up to 20 days. Maintenance of acinar cells for longer periods for progressive studies is essential to understand the exocrine pancreatic biology. The medium used in this study was supplemented with EGF and BSA; EGF was added in order to promote growth and proliferation of cells and BSA was used to protect the cells from premature activation of protease (especially trypsin), the activator of other zymogens [30]. These supplements possibly could have played a vital role in maintaining the cells' functionality for a longer period. This observation leads to the speculation that an appropriately enriched nutritional medium might be helpful in prolonging the viability and effective functionality in general and acinar cells in particular.

The activity profile of the enzymes secreted by cultured cells showed high level of protease, followed by lipase and then amylase. Gradual decrease or absence of increase in activities of these enzymes was observed after the 15th day of culture. This may be due to the depletion of micro-environmental requirements necessary for cell survival and enzyme secretions.

Pancreatic tissue consists of several cell types including acinar and β as major constituent cells. Each cell type is identified based on its own characteristic secretory products. Among the enzymes secreted by acinar cells, amylase is used as a marker for immunofluorescence study. The production and activity of this enzyme pronounces the presence of acinar cells in functional state. The fluorescence emitted by amylase antibody bound cells indicates that the cell population exclusively comprised of acinar cells, the identity of which was confirmed by its amylase producing property. These observations are in accordance with a previous study revealing that the acinar cells are positive for amylase [31]. This antibody is expected to bind with an epitope corresponding to amino acids 212–492 of human pancreatic amylase 2B. Even in this study, the human antibody was able to bind with bovine pancreatic acinar cells and emit red fluorescence indicating not only the purity of acinar culture (Fig. 3A) but also indicates that both human and bovine amylase share a common epitope. This finding is in good agreement with the report of Tellam and his co-workers [32] who sequenced the whole bovine genome and compared it with human genome. Their findings revealed that there was an 80% sequence similarity between bovine and human genomes. They also stated that bovine proteins show more commonality compared to that of rodents. With this idea and the observation of immunofluorescence study, we have done sequence similarity search using “The Basic Local Alignment Search Tool for proteins (BLAST)” for amylase 2B protein of bovine and human and found that there was 86% identity, which is similar to pigs. This leads to a notion that bovine cells also could be used as an alternative for pigs for human biomedical applications.

Examination of cells at high magnification employing electron microscopy as a tool had a remarkable impact on cell biology [33], as this examination aids in visualizing both the surface and ultra-structure of the cells. The secretory granule is spherical and surrounded by an intact, thick bi-layered membrane when the ultra-structure is visualized under TEM. This lipid bilayer plays a crucial role in pathology of pancreas, because if the layer gets digested and allows the entry of enzyme activators it may lead to the activation of trypsin and damage the whole pancreas itself. Therefore this bi-layer acts as a barrier and shelters the granules from the access of other modifying enzymes and is associated with several proteins which represent important components in the regulated secretion of digestive enzymes [34]. Hence the secretory granule is designed in such a way that it is inaccessible to enzymes. Chen and his co-workers [35] studied the molecular topology of zymogen granule and reported that there are hundreds of proteins present in the lipid bilayer which helps in preventing the enzyme attack and one of the proteins v-SNARE (soluble N-ethylmaleimide-sensitive factor attachment protein receptors of vesicle) is involved in fusing with t-SNARE (of target membrane) of plasma membrane during exocytosis. The exocytosis process is triggered by a cascade of cellular events which leads to the generation of intracellular messengers. The granules after release from golgi complex get trafficked near plasma membrane. The trafficking of secretory granules to the plasma membrane of eukaryotic cells is essential for normal cellular function. It also forms the basis of intercellular communication in multicellular organisms through release of a wide array of extracellular acting molecules [36].

In this study, we were able to observe the trafficked secretory granules and the fusion pore/porosome formed on plasma membrane of the cell in SEM as well as in TEM. The secretory granule releases its contents through these porosomes and was first discovered in acinar cells of the rat pancreas nearly 17 years ago [37].

The enzymes released as a result of this fusion complex and the resulted fusion pores were observed in SEM. The granules released from the cell membrane adhere to the surface and are non-homologous in nature, with different shapes and sizes, which is similar to the observation of Bendayan et al. [13] under TEM. The non-homologous nature of the secretory products may be due to the presence of different kinds of digestive enzymes with varying concentration. The enzymes are secreted by exocytosis in a constitute manner *via* basal secretion by the cultured acinar cells.

In summary, the cells isolated from bovine pancreas could grow well *in vitro,* and were able to secrete enzymes and pack them up in lipid bilayer protected secretory granules, respond to signal and communicate *via* intracellular molecules to dock and fuse with plasma membrane in order to release the packed enzymes. Further research on this culture model is now in progress to investigate the response of cultured cells upon stimulation using secretagogue to study the pathogenesis of pancreas and the signalling pathway involved in exocytosis.
