# Supplementary material for: In vitro secretion of zymogens by bovine pancreatic acini and ultra-structural analysis of exocytosis
Source: Biochem Biophys Rep. 2015 Dec 23;5:237–45. doi: 10.1016/j.bbrep.2015.12.009 (PMC5600341; doi:10.1016/j.bbrep.2015.12.009)
Supplement: Supplementary file 1 — Supplementary material [file mmc1.doc]

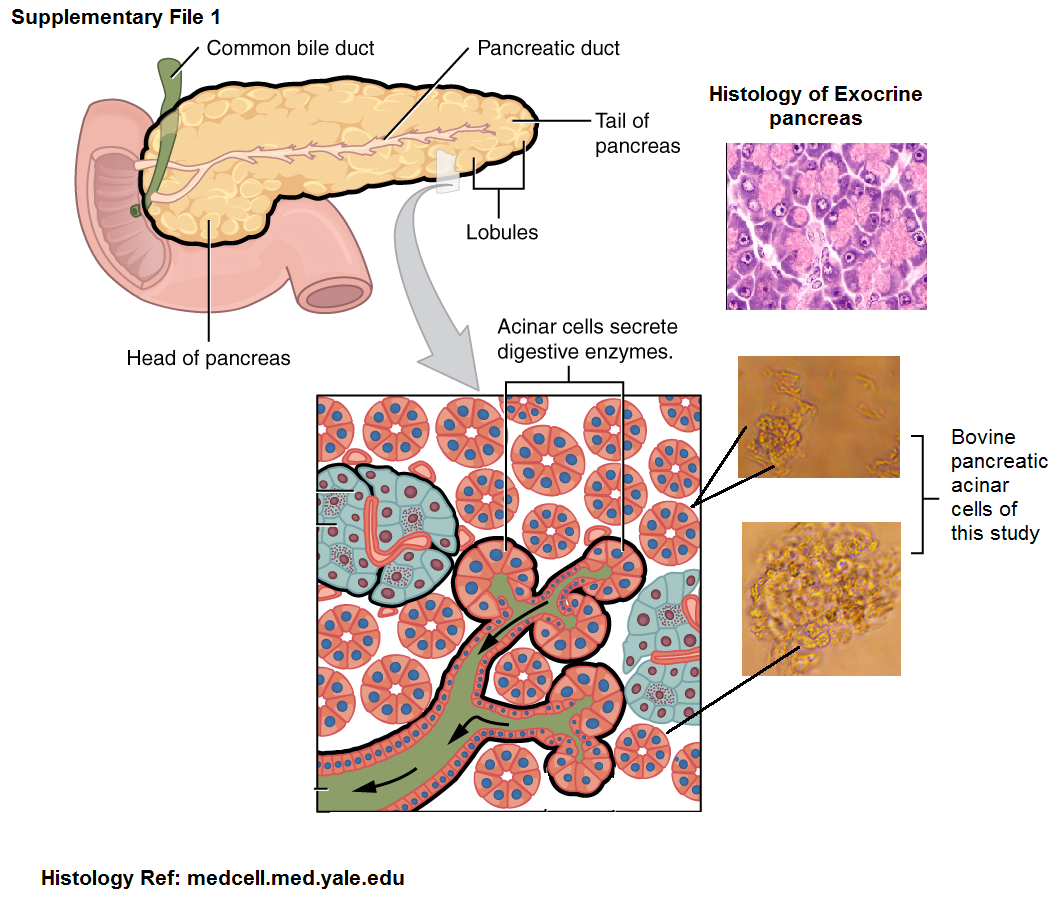


**Fig.S1: Structural organization of acini *in vitro*** **and *in vivo* defining the structural similarity between isolated cells and the tissue**


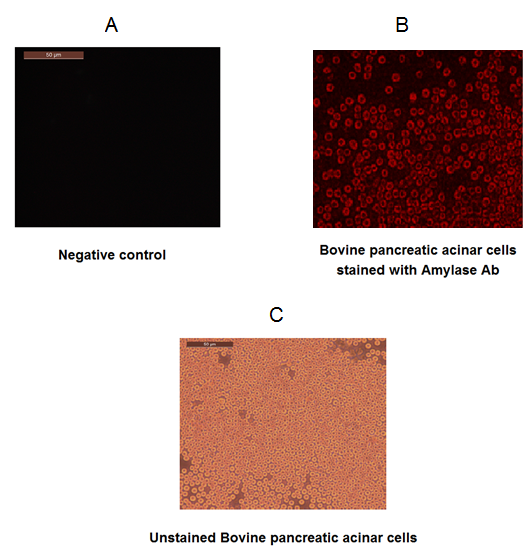


**Fig.S2: Cytoplasmic localization of amylase in bovine pancreatic acini. a) Neagtive control b) Stained cells c) unstained cells**


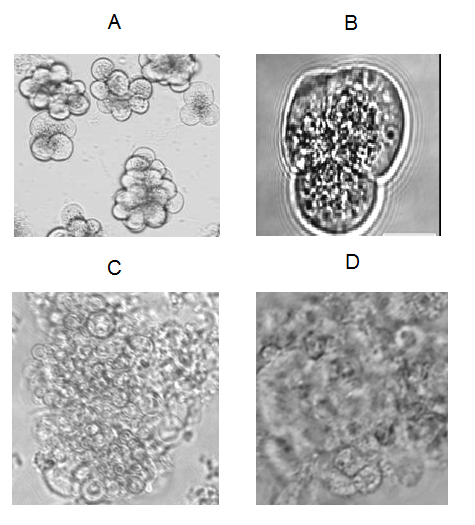


**Fig.S3: Phenotypic comparison of acini isolated from pancreas of (A) rat (B) Human**

**(C) & D) bovine (cells of this study)**

**A) Ref : Williams, John A. (2010), Pancreapedia: Exocrine Pancreas Knowledge Base, DOI: 10.3998/panc.2010.18**

**B) Cane, Matthew C., Sutton, Robert, and Criddle, David N. (2011)** [**Pancreapedia: Exocrine Pancreas Knowledge Base**](http://www.pancreapedia.org/)**, DOI:**[**10.3998/panc.2011.20**](http://dx.doi.org/10.3998/panc.2011.20)
