# Supplementary material for: In vitro secretion of zymogens by bovine pancreatic acini and ultra-structural analysis of exocytosis
Source: Biochem Biophys Rep. 2015 Dec 23;5:237–45. doi: 10.1016/j.bbrep.2015.12.009 (PMC5600341; doi:10.1016/j.bbrep.2015.12.009)

AUTHOR DECLARATION

We wish to confirm that there are no known conflicts of interest associated with this publication.We confirm that the manuscript has been read and approved by all named authors and that there are no other persons who satisfied the criteria for authorship but are not listed. We further confirm that the order of authors listed in the manuscript has been approved by all of us.

We understand that the Corresponding Author is the sole contact for the Editorial process (including Editorial Manager and direct communications with the office). He is responsible for communicating with the other authors about progress, submissions of revisions and final approval of proofs. We confirm that we have provided a current, correct email address which is accessible by the Corresponding Author and which has been configured to accept email from (chellanrose@yahoo.co.uk)

CHELLAN ROSE 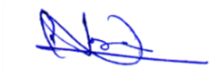

JAYAVENI SIVALINGAM 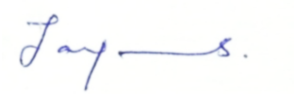

NITHYANANDHAM KAMARAJ 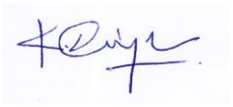

Supplement: Supplementary file 2 — Supplementary material [file mmc2.pdf]
